# Chicago Veterinary Society

**Published:** 1897-03

**Authors:** Lawrence Campbell

**Affiliations:** Secretary


Secretary.

### CHICAGO VETERINARY SOCIETY.

Meeting called to order February 11, 1897, by the President, Dr. Walker. Owing to the tardiness of many of the members it was 8.45 P.M. before the meeting was called to order. On motion, roll-call was dispensed with, a count showing only fifteen members present. The Secretary read the minutes of the two previous meetings, which were approved. Moved by Dr. Campbell, seconded by Dr. Worms, to dispense with the regular business and go on to the admission of new members. After the reading of the applications by the Secretary and the approval of the same by the Board of Censors, the following gentlemen were elected to membership by acclamation: Dr. Frank McCoy, Dr. Harry J. Stewart, Dr. J. A. Bovett, Jr.

A copy of a letter[1] which some time ago was sent to the Boss Horseshoers' Association, was then read to the Society. The latter was originally sent by a committee of three members. The Boss Horseshoers never having acknowledged the letter, it was moved and seconded to have the Secretary send them a copy of the letter, with another letter, asking them to acknowledge the same and give the Society their views on the matter. The letter referred to the practice of veterinary medicine and surgery by the horseshoers, and asking them not to do so.

The next order of business being new business, a lobbyist was introduced to the Society, who spoke about the Illinois State veterinary bill, now pending before the Legislature, at Springfield. Motion by Dr. Hughes, seconded by Dr. Worms, that three members be appointed to go to Springfield on the 16th to interview the representatives and to try to further the interests of the State bill. The President asked Dr. Hughes to appoint the committee. Dr. Hughes appointed Dr. James Henderson, Dr. E. L. Quitman, and Dr. Campbell, and stated that either he or Dr. Baker would also go down to represent the Chicago Veterinary College. Dr. Walker stated that he would also try to go.

Dr. A. H. Baker was the essayist of the evening, but owing to sickness he was unable to read his paper on "Open Joint." The paper was deferred until the next meeting. There being no further business, Dr. Hughes presented to the Society the *four* feet of a subject which had come under his notice; the peculiarity of the feet were that they were *all* affected with navicular disease. Dr. Hughes exhibited the feet (showing the navicular bone) because it was so unusual to find an animal affected with this disease both in front and behind. Quite a discussion was held on the subject, and quite an instructive talk was given by Dr. Hughes. A vote of thanks was given to Dr. Hughes for the exhibition of such an unusual case, and for his interesting discourse on the same.

On motion, adjournment. LAWRENCE CAMPBELL,
Secretary.

[1] Will be published in April number.

